# Cocaine-Induced Locomotor Activation Differs Across Inbred Mouse Substrains

**DOI:** 10.3389/fpsyt.2022.800245

**Published:** 2022-05-06

**Authors:** Christiann H. Gaines, Sarah A. Schoenrock, Joseph Farrington, David F. Lee, Lucas J. Aponte-Collazo, Ginger D. Shaw, Darla R. Miller, Martin T. Ferris, Fernando Pardo-Manuel de Villena, Lisa M. Tarantino

**Affiliations:** ^1^Department of Genetics, School of Medicine, University of North Carolina at Chapel Hill, Chapel Hill, NC, United States; ^2^Neuroscience Curriculum, University of North Carolina at Chapel Hill, Chapel Hill, NC, United States; ^3^Pharmacology Curriculum, University of North Carolina at Chapel Hill, Chapel Hill, NC, United States; ^4^Department of Pharmacology, School of Medicine, University of North Carolina at Chapel Hill, Chapel Hill, NC, United States; ^5^Lineberger Comprehensive Cancer Center, School of Medicine, University of North Carolina at Chapel Hill, Chapel Hill, NC, United States; ^6^Division of Pharmacotherapy and Experimental Therapeutics, Eshelman School of Pharmacy, University of North Carolina at Chapel Hill, Chapel Hill, NC, United States

**Keywords:** cocaine sensitivity, initial cocaine response, genetics, reduced complexity cross, rodent behavior, addiction, rodent model, mice models

## Abstract

Cocaine use disorders (CUD) are devastating for affected individuals and impose a significant societal burden, but there are currently no FDA-approved therapies. The development of novel and effective treatments has been hindered by substantial gaps in our knowledge about the etiology of these disorders. The risk for developing a CUD is influenced by genetics, the environment and complex interactions between the two. Identifying specific genes and environmental risk factors that increase CUD risk would provide an avenue for the development of novel treatments. Rodent models of addiction-relevant behaviors have been a valuable tool for studying the genetics of behavioral responses to drugs of abuse. Traditional genetic mapping using genetically and phenotypically divergent inbred mice has been successful in identifying numerous chromosomal regions that influence addiction-relevant behaviors, but these strategies rarely result in identification of the causal gene or genetic variant. To overcome this challenge, reduced complexity crosses (RCC) between closely related inbred mouse strains have been proposed as a method for rapidly identifying and validating functional variants. The RCC approach is dependent on identifying phenotypic differences between substrains. To date, however, the study of addiction-relevant behaviors has been limited to very few sets of substrains, mostly comprising the C57BL/6 lineage. The present study expands upon the current literature to assess cocaine-induced locomotor activation in 20 inbred mouse substrains representing six inbred strain lineages (A/J, BALB/c, FVB/N, C3H/He, DBA/2 and NOD) that were either bred in-house or supplied directly by a commercial vendor. To our knowledge, we are the first to identify significant differences in cocaine-induced locomotor response in several of these inbred substrains. The identification of substrain differences allows for the initiation of RCC populations to more rapidly identify specific genetic variants associated with acute cocaine response. The observation of behavioral profiles that differ between mice generated in-house and those that are vendor-supplied also presents an opportunity to investigate the influence of environmental factors on cocaine-induced locomotor activity.

## Introduction

Recent data indicate that cocaine use, the prevalence of cocaine use disorder (CUD) and cocaine-related overdose deaths have been increasing in the United States (1–4). Although cocaine use and abuse remain a significant public health concern, there are currently no FDA approved therapies for CUD. The lack of treatment options is due, in part, to gaps in our knowledge about the etiology of this complex and devastating disorder.

Not all who use cocaine will go on to develop a CUD, suggesting that individual differences contribute to risk. Twin studies yield heritability estimates of approximately 0.70 for cocaine dependence indicating a significant genetic contribution to risk of developing a CUD (5). CUD risk is also heavily influenced by the environment and gene by environment interactions (5–9). Human genome wide association studies (GWAS) have been successful in identifying specific loci and genes associated with nicotine dependence and alcohol use disorders (10–13). The few GWAS studies that have been published for cocaine dependence or CUD have suffered from insufficient sample sizes, limiting discovery of loci and genes that contribute to CUD risk (14–16). Identifying genetic and molecular pathways implicated in CUD would provide insight into individuals at increased risk and generate novel targets that could be investigated for development of effective therapeutics.

Genetic mapping studies and follow-up of loci identified in human GWAS using rodent models provides complementary approaches to human GWAS studies of CUD. One notable example is the identification of the family with sequence similarity 53, member B (*FAM53B*) gene as a risk variant for cocaine dependence in a human CUD GWAS and a mouse mapping study of self-administration of cocaine (14, 17). The use of rodent models offers several advantages, including the ability for the genetic background, environment, and drug exposure regimens to be controlled and manipulated. While rodent models cannot fully recapitulate the range of symptoms observed in human CUD, they do allow for measurement of specific addiction-relevant behaviors, including initial drug sensitivity. Retrospective and longitudinal studies in humans have shown that individual differences in initial subjective drug responses can predict subsequent drug use (18–21). In mice, acute locomotor response to an initial dose of cocaine is a well-established model of initial sensitivity (22, 23).

Genetic mapping studies in inbred mouse strains have successfully identified genomic regions, termed quantitative trait loci (QTL), that are associated with cocaine-induced locomotor activation (17, 24–30). Traditional mapping approaches typically involve crossing genetically and phenotypically diverse pairs of inbred strains and intercrossing or backcrossing the resulting F1s to generate F2 or N2 mapping populations, respectively. The resulting QTL identified in these studies typically span tens of megabases containing hundreds of genes and thousands of potential causal polymorphisms. Therefore, identifying the specific variant(s) that affect cocaine-induced locomotor activation and other complex behavioral traits has been extremely challenging.

Reduced Complexity Crosses (RCC) between inbred mouse substrains offer a significant advantage over traditional genetic mapping strategies. Substrains are nearly isogenic inbred strains derived from the same founder strain that have been bred independently for multiple generations (typically >20). An RCC is generated in the same fashion as an F2 or N2 population described above, by crossing two substrains that differ for a phenotype of interest. QTL identified in RCCs are similarly sized in comparison to those identified using traditional F2 mapping populations, but causal polymorphisms in the region are limited to those that were still segregating at the time the strains were separated or arose spontaneously since that time (31, 32). This feature of RCCs dramatically facilitates detection of polymorphisms within the QTL region and identification of the causative polymorphism (32). Additionally, a recently developed genotyping array captures polymorphisms between inbred mouse substrains, facilitating rapid and reliable genotyping of RCCs (33). RCCs have been used successfully to identify genetic polymorphisms that impact psychostimulant response, binge eating, binge alcohol consumption, thermal nociception and brain weight (34–38).

Genetic differences between substrains are likely to influence any number of phenotypes, offering a powerful tool with which to expand our knowledge about the genetic loci that affect addiction-relevant behaviors. However, the literature describing substrain differences in locomotor response to drugs of abuse has been limited primarily to the C57BL/6 substrains. In this study, we measured cocaine-induced locomotor activation across 20 substrains derived from A/J, BALB/c, DBA/2, FVB/N, NOD and C3H/He inbred mouse strains. We report significant substrain differences in acute cocaine locomotor activation in response to an acute exposure to 20 mg/kg cocaine. Our data significantly expand the current knowledge about substrain differences in cocaine locomotor response and offer the opportunity to pursue genetic studies to identify genes that contribute to this behavior.

## Materials and Methods

### General Methods

Mice were all housed in a pathogen-free facility at UNC. This facility consisted of a 12-h light/dark cycle with lights on at 7:00 AM. All animal care and protocols were approved by the University of North Carolina at Chapel Hill (UNC) Institutional Animal Care and Use Committee and followed guidelines that were implemented by the National Institutes of Health Guide for the Care and Use of Laboratory Animals, 8th Edition. Mice were maintained in AAALAC-accredited, specific pathogen free (SPF) barrier colony in ventilated cages (Tecniplast, Buguggiate, Italy). Food (PicoLab Rodent Diet 20, Purina, St. Louis, Missouri) was provided *ad libitum* and throughout the duration of behavioral testing. Edstrom carbon filtered; reverse osmosis hyper-chlorinated water was provided *ad libitum* except during behavioral testing.

Two groups of mice were used for behavioral testing (see Supplementary Table 1 for a summary of substrains, origin, housing, and vendor). The first group consisted of six sets of substrains that were originally purchased from their respective vendors and bred in the vivarium at UNC. Mice bred at UNC were either group-housed with cagemates of the same substrain or co-housed at weaning (postnatal day 21) with mice from other substrains within their strain group (i.e. DBA/2J, DBA/2NCrl and DBA/2NTac mice in the same cage). The second group consisted of four sets of substrains that were purchased directly from their respective commercial vendors, delivered to our vivarium at 6–7 weeks of age and maintained in group housing throughout testing. Since these substrains were received close to testing age, they were maintained in substrain-specific cages and not co-housed due to concerns about aggressive behavior among males that had not been previously co-housed.

Vendor supplied substrains were an average age of 62 days old at the start of testing. Mice bred in-house were an average age of 65 days old at the start of testing. All mice were weighed on the day prior to testing and weights were used to determine the volume of saline or cocaine administered during testing. Mice were transported to the procedure room located within the same vivarium immediately prior to the start of testing. Behavioral testing occurred during the light cycle from 8:00 AM to 12:00 PM with the time that a mouse was tested being consistent across the three test days. All vendor-supplied substrains were tested by the same experimenter (female) whereas those bred in-house were tested by 5 different experimenters (male and female).

### Vendor Supplied Substrains

Information on all inbred substrains including source and cage environment (cohoused vs not cohoused) is provided in Supplementary Table 1. A/J, BALB/c, FVB/N and DBA/2 substrains were purchased from their respective commercial vendors and housed in substrain specific cages throughout testing. Mice were an average of 27 days old upon arrival to UNC and were acclimated to the vivarium for 5 weeks after arrival before behavioral testing. A/J substrains were A/J (The Jackson Laboratory, 000646), A/JCr (Charles River Laboratories, 563) and A/JOlaHsd (Envigo, 049). BALB/c substrains were BALB/cJ (The Jackson Laboratory, 000651), BALB/cByJ (The Jackson Laboratory, 001026), BALB/cAnNCrl (Charles River Laboratories, 028) and BALB/cAnNHsd (Envigo, 047). FVB/N substrains were FVB/NJ (The Jackson Laboratory, 001800), FVB/NCrl (Charles River Laboratories, 207), FVB/NHsd (Envigo, 118), and FVB/NTac (Taconic Biosciences, FVB-F/FVB-M). DBA/2 substrains were DBA/2J (The Jackson Laboratory, 000671), DBA/2NCrl (Charles River Laboratories, 026) and DBA/2NTac (Taconic Biosciences, DBA2-F/DBA2-M).

### Substrains Bred In-house

Another cohort of inbred mouse substrains were purchased from commercial vendors but test animals were bred in-house at UNC. The following substrains were tested in this cohort: DBA/2J, DBA/2NCrl, DBA/2NTac, A/J, A/JOlaHsd, BALB/cByJ, BALB/cJ, FVB/NJ, FVB/NTac, NOD/MrkTac (Taconic Biosciences, NOD-F/NOD-M), NOD/ShiLtJ (The Jackson Laboratory, #001976), C3H/HeJ (The Jackson Laboratory, #000659), C3H/HeNTac (Taconic Biosciences, C3H-F/C3H-M), C3H/HeNHsd (Envigo, 040) and C3H/HeNCrl (Charles River Laboratories, 025). Some of these mice were cohoused with cagemates that included at least two different substrains from a single progenitor strain.

### Drugs

Cocaine hydrochloride (HCl) was purchased from Sigma-Aldrich (St. Louis, MO, USA; C5776-5G). A solution of cocaine HCl was prepared fresh daily. Cocaine HCl was dissolved in physiological saline at a concentration of 2 mg/ml and administered via intraperitoneal (i.p.) injection at a volume of 0.01 ml/g resulting in a dose of 20 mg/kg of body weight administered to mice for behavioral testing. Saline was purchased from Fisher Scientific (Waltham, MA, USA; 297753).

### Open Field Apparatus

The open field (OF) arena (ENV-515-16, Med Associates, St. Albans, VT, USA), measured 17 x 17 x 13cm and consisted of four clear Plexiglas walls and a white Plexiglas floor. The walls are surrounded by infrared detection beams on the X, Y, and Z axes used to detect horizontal and vertical activity of the animal throughout the duration of the test session. The OF chamber is placed within a sound attenuating box (73.5 x 59 x 59 cm) that has two overhead light fixtures containing 28-V lamps. Light levels on the arena floor were 24 lux in the center, 10 lux in the corners and 13 lux along the walls. Eight identical OF arenas were used for testing with a mouse being tested in the same arena each test day.

### Acute Cocaine-Induced Locomotor Activity Test

On Day 1, behaviorally naïve mice were given an i.p. injection of saline at a volume of 0.01 ml/g body weight and immediately placed into the OF for 30 min to habituate to the arena. On Day 2, mice were again given an i.p. injection of saline and placed into the OF for 30 min. On Day 3, mice were given an i.p. injection of 20 mg/kg of cocaine and placed into the OF chamber for 30 min. Locomotor behavior was measured as total distance moved (in centimeters) for the entire 30-min test period each day using the manufacturers data acquisition software (Activity Monitor v5.9.725; Med Associates). Locomotor activity recorded during day 2 was used as a baseline measurement for comparison with cocaine-induced locomotor activity on Day 3. At the end of each test session, mice were placed back into their home cages and the OF chambers were cleaned with 0.25% bleach solution.

### Statistical Analyses

All statistical analyses were performed using SPSS v28 for Mac (IBM Inc). Due to normality of data variables, a Box-Cox transformation of locomotor activity data for each substrain was performed and the resulting transformation used for each strain group is reported in Supplementary Table 2. For each set of substrains, we performed an ANOVA that included day of testing, substrain and sex as independent variables and locomotor activity as the dependent variable. In cases where multiple individuals tested animals within the same strain cohorts, we added experimenter as a covariate in the ANOVA model. Significant main effects (*p* < 0.05) were followed up with *post-hoc* Tukey's HSD or independent samples T-tests.

## Results

Experimental data for all inbred mouse substrains including origin of the mice, number of mice tested, cage environment, strain means and standard deviations are provided in Supplementary Table 1. We observed significant substrain differences in basal and/or cocaine-induced locomotor activity in all 6 strain groups we examined (Supplementary Table 2). C3H/He and DBA/2 substrain differences were fairly stable across experimental cohorts. We also observed substrain differences (i.e., A/J and FVB/N) that were not replicated across experimental groups (vendor-supplied vs in-house). Sex differences also varied across and within strain groups and experimental cohorts. Results presented individually by substrain are described below.

### A/J Substrains Are Not Activated Upon Acute Exposure to 20 mg/kg Cocaine

#### Vendor Supplied

**Figure 1:**
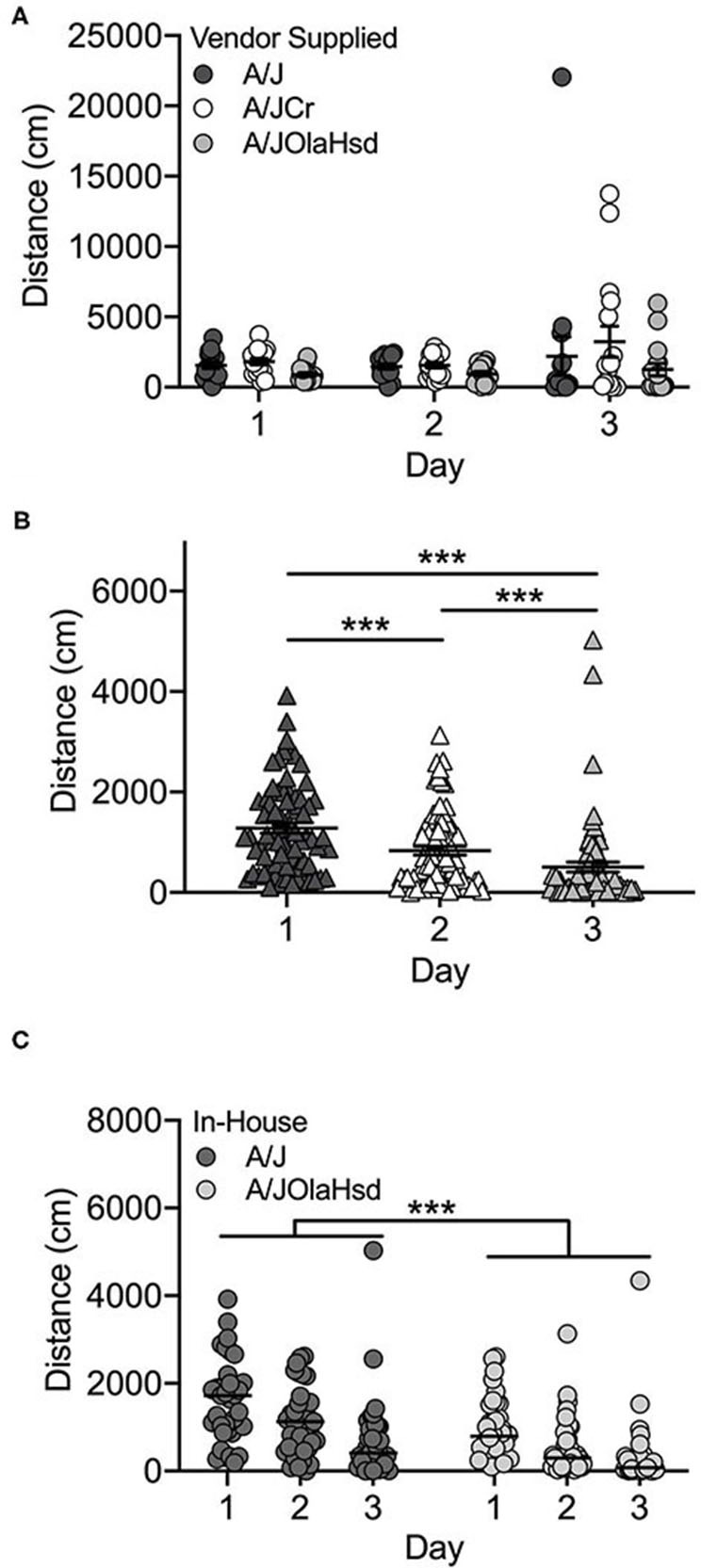
A/J substrains are not activated in response to cocaine. **(A)** Vendor-supplied A/J substrains do not exhibit increased activity in response to cocaine on Day 3; **(B)** locomotor activity decreases across all three days of testing in A/J and A/JOlaHsd substrains bred in-house and **(C)** A/J mice are significantly more active than A/JOlaHsd mice regardless of day. Each data point represents an individual mouse, error bars are SEM. ***p < 0.001.

Overall, the locomotor activity of the vendor-supplied A/J substrains did not increase significantly after exposure to cocaine (F_(2, 126)_ = 0.17; *p* = 0.846; Figure 1A), although increased cocaine-induced locomotor activity can be observed in the A/JCr substrain. There was a significant main effect of substrain (F_(2, 126)_ = 4.1; *p* = 0.019). A/JCr mice were significantly more active than A/JOlaHsd mice (*p* = 0.014), but this appears to be mostly driven by several high-responding A/JCr mice (data not shown). No significant sex (F_(1, 126)_ = 0.73; *p* = 0.396) or interaction effects were observed.

#### Bred In-house

Two A/J substrains were bred in-house at UNC for 1–2 (A/JOlaHsd) or 3-4 (A/J) generations. These mice showed a different behavioral profile than those obtained from commercial vendors. We observed a significant decrease in locomotor activity across all 3 days, including Day 3 after exposure to cocaine (Figure 1B; F_(2, 206)_ = 30.5; *p* = 2.5 x 10^−12^). We also observed significant substrain (F_(1, 206)_ = 34.9; *p* = 1.4 x 10^−8^) and sex [F_(1, 206)_ = 7.1; *p* = 0.009] effects. The A/J substrain showed significantly higher locomotor activity regardless of day (*t*(217) = 5.2; *p* = 4.1 x 10^−7^; Figure 1C). Overall, male mice were significantly more active than female mice (*t*(217) = 2.5; *p* = 0.013; data not shown). There were no significant interactions among any of the independent variables tested.

### Locomotor Response to Cocaine Differs in BALB/c Substrains From the “J” Lineage in Comparison With Substrains From the “AnN” Lineage

#### Vendor Supplied

**Figure 2:**
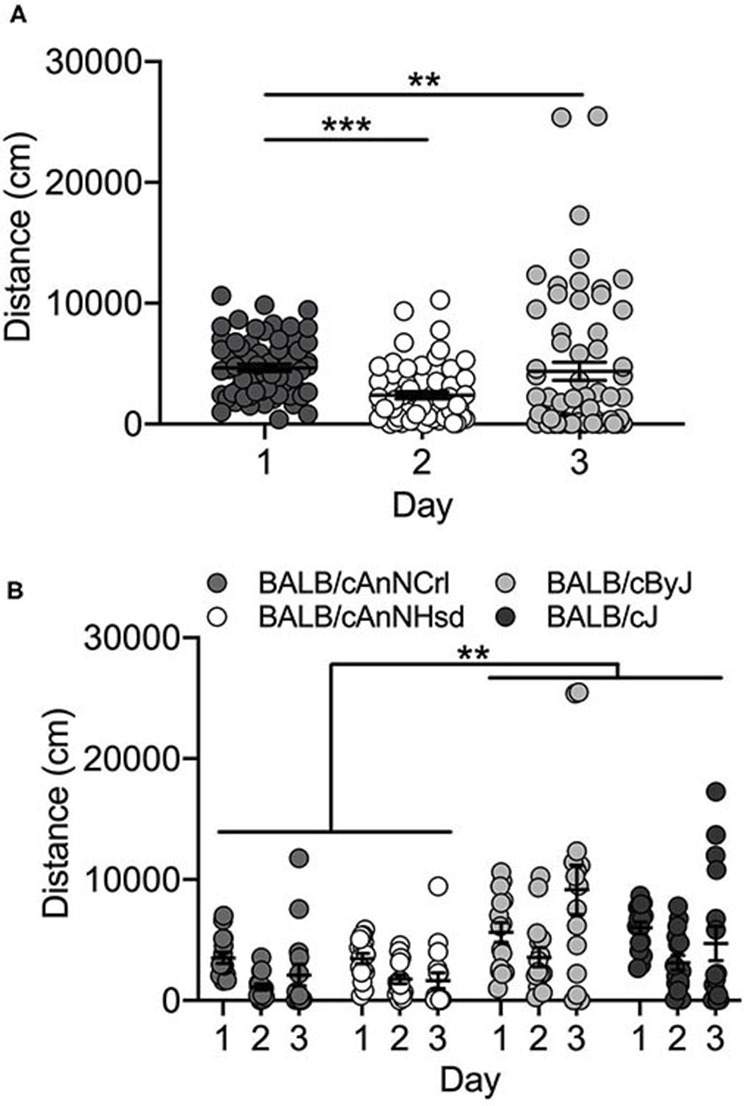
BALB/c substrains are not activated in response to cocaine. **(A)** Regardless of substrain, locomotor activity was significantly higher on Day 1 in response to saline in comparison to Day 2 activity (saline) and Day 3 cocaine-induced locomotor activity. **(B)** BALB/c substrains were significantly more active than BALB/cAn substrains across all three days. Each data point represents an individual mouse, error bars are standard error of the mean. **p < 0.01; ***p < 0.001.

Vendor-supplied BALB/c substrains showed no locomotor activation in response to cocaine exposure on Day 3. Rather, locomotor activity decreased across the three days of testing in the four substrains [F_(2, 162)_ = 10.8; *p* = 3.9 x 10^−5^]. Collapsed across substrains, locomotor activity on Day 1 is significantly higher than either Day 2 (*p* = 3.7 x 10^−5^) or Day 3 (*p* = 0.004) (Figure 2A). We also observed a significant main effect of substrain [F_(3, 162)_ = 13.6; *p* = 6.2 x 10^−8^]. Mice of both J substrains (BALB/cJ and BALB/cByJ) are significantly more active than both BALB/cAnNHsd and BALB/cAnNCrl mice (all *p* < 0.01; Figure 2B). Female mice were significantly more active than male mice [F_(1, 162)_ = 5.9; *p* = 0.016; data not shown].

#### Bred In-house

BALB/cJ and BALB/cByJ mice were generated in-house and we tested offspring from the 1st and 2nd generations of breeding from the initial vendor stock. We observed no significant day [F_(2, 27)_ = 2.4; *p* = 0.106] or substrain [F_(1, 27)_ = 0.12; *p* = 0.731] differences. We did observe a sex difference [F_(1, 27)_ = 4.5; *p* = 0.043], but it should be noted that our experimental cohort was limited to only 2 BALB/cByJ males and no BALB/cJ males (data not shown).

### C3H/HeNTac Mice Are More Active Than Other C3H/He Substrains

#### Bred In-house

All C3H/He substrains were bred in-house. The initial cohort was limited to C3H/HeJ and C3H/HeNTac substrains that were the first generation of offspring from vendor-supplied mice (C3H/HeJ) or offspring of crosses between mice from the third generation bred at UNC (C3H/HeNTac). These animals were cohoused such that mice from each substrain were weaned into cages together and maintained in that manner throughout testing. The second cohort of mice included C3H/HeNCrl and C3H/HeNHsd substrains in addition to C3H/HeNTac and C3H/HeJ, and were produced by breeding vendor-supplied mice at UNC for one generation. These mice were weaned into and maintained in substrain-specific caging throughout testing.

**Figure 3:**
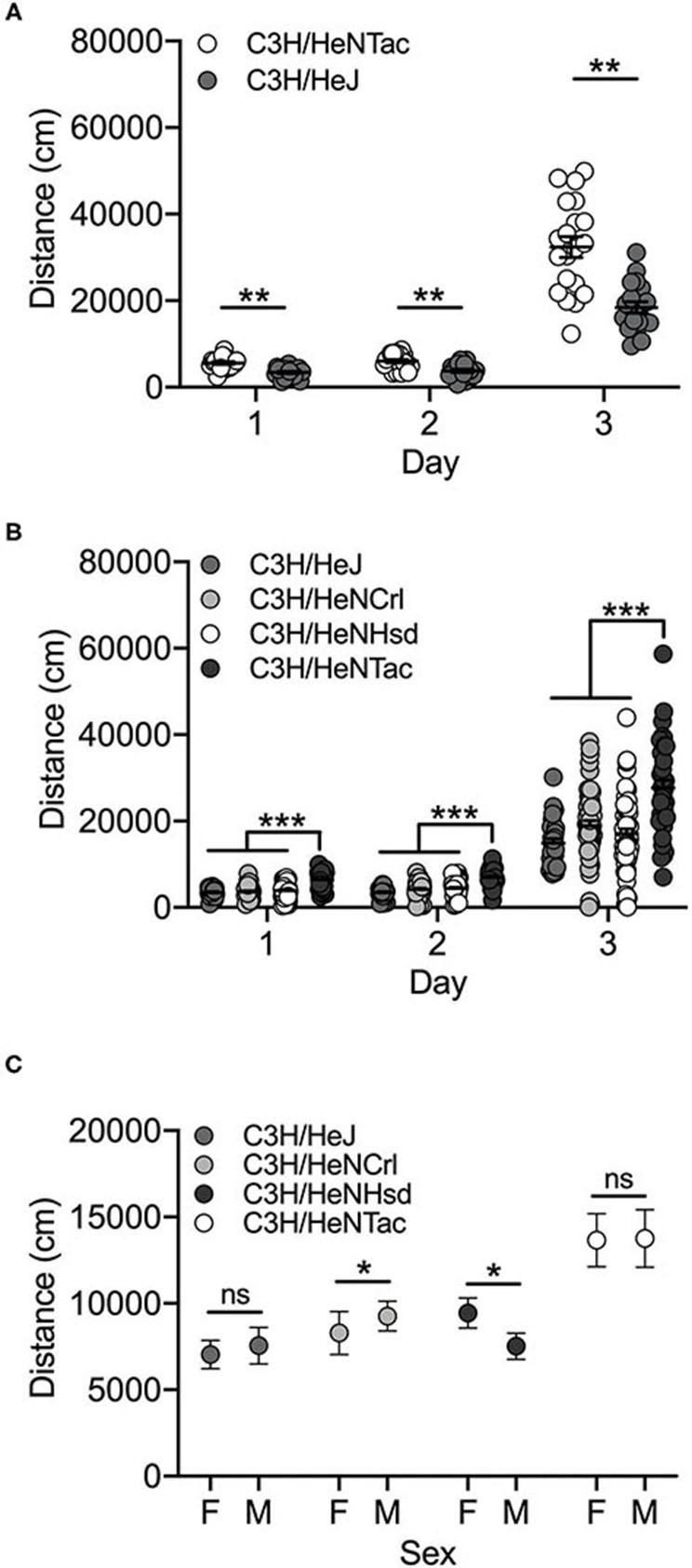
C3H/HeNTac mice are more active than other C3H/He substrains. **(A)** Cohoused C3H/HeJ and C3H/HeNTac substrains are significantly activated by cocaine on Day 3 and C3H/HeNTac mice are significantly more active than C3H/HeJ mice regardless of treatment (saline, cocaine). **(B)** Non-cohoused C3H/He substrains are significantly activated in response to cocaine on Day 3 and C3H/HeNTac mice are significantly more active than C3H/HeJ, C3H/HeNCrl and C3H/HeNHsd substrains. **(C)** C3H/HeNCrl males are significantly more active than females and C3H/HeNHsd females are significantly more active than males. Each data point represents an individual mouse, error bars are standard error of the mean. *p < 0.05; **p < 0.01; ***p < 0.001.

Both C3H/HeJ and C3H/HeNTac substrains in the cohoused cohort were significantly more active in response to cocaine (Day 3) vs saline (Days 1 and 2) [F_(2, 104)_ = 246.3; *p* = 3.6 x 10^−40^]. We also observed a significant substrain effect [F_(1, 104)_ = 52.5; *p* = 7.9 x 10^−11^]. C3H/HeNTac mice were significantly more active than C3H/HeJ mice (*t*(115) = 3.3; *p* = 0.001; Figure 3A). No sex differences [F_(1, 104)_ = 0.0003; *p* = 0.986] or interaction effects were observed.

In the non-cohoused cohort comparing all four C3H/He substrains, we observed a significant effect of substrain [F_(3, 527)_ = 27.0; *p* = 3.0 x 10^−16^] and day [F_(2, 527)_ = 277.5; *p* = 4.7 x 10^−83^]. Cocaine-induced locomotor activity on Day 3 was significantly higher than activity on Days 1 and 2 (both *p* < 0.001). As in the cohoused cohort, C3H/HeNTac mice were significantly more active than C3H/HeJ (*p* = 2.3 x 10^−12^) and both C3H/HeNCrl (*p* = 1.2 x 10^−12^) and C3H/HeNHsd (*p* = 1.3 x 10^−12^) substrains (Figure 3B; all *p* < 0.001). We also observed a significant substrain by sex interaction; C3H/HeNCrl males are significantly more active than females (*p* = 0.013) whereas the opposite is true for C3H/HeNHsd (*p* = 0.01). Two different individuals tested mice in this cohort and we identified experimenter as a significant covariate (*p* = 2.0 x 10^−6^). The experimenter effect reflects higher locomotor activity across all substrains following cocaine administration on Day 3 in mice tested by one experimenter vs the other (data not shown).

The availability of data from both cohoused and non-cohoused C3H/HeJ and C3H/HeNTac mice allowed us to examine the effects of housing on behavior in these two substrains. An ANOVA including housing (cohoused vs. non-cohoused) as well as day, strain and sex as independent variables yielded no significant main effect of housing [F_(1, 300)_ = 2.4; *p* = 0.120]. A significant housing by day interaction [F_(2, 300)_ = 4.9; *p* = 0.008] suggested that non-cohoused mice were significantly less active than cohoused mice in response to an acute exposure to cocaine on Day 3, but *post hoc* tests revealed no significant difference (data not shown).

### DBA/2NTac Mice Were Significantly Less Active Than DBA/2J and DBA/2NCrl Mice

#### Vendor Supplied

**Figure 4:**
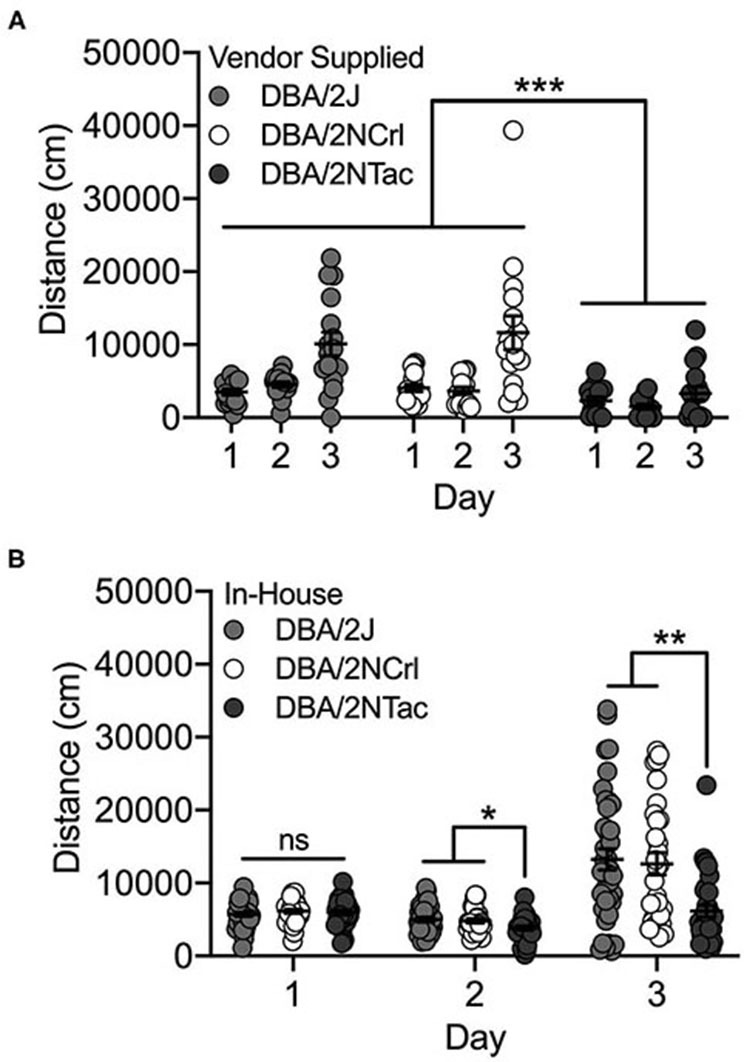
DBA/2NTac mice are significantly less active than DBA/2J and DBA/2NCrl mice. **(A)** All vendor-supplied DBA/2 substrains are significantly more active in response to cocaine on Day 3 vs saline on Days 1 and 2. DBA/2NTac mice are significantly less active than mice from both DBA/2J and DBA/2NCrl substrains regardless of day. **(B)** DBA/2 substrains bred in-house are also significantly more active in response to cocaine on Day 3 compared with saline on Days 1 and 2. DBA/2NTac mice are significantly less active than DBA/2J and DBA/2NCrl mice on Days 2 and 3, but not on Day 1. Each data point represents an individual mouse, error bars are standard error of the mean. *p < 0.05, **p < 0.01, ***p < 0.001.

We observed significant day [F_(2, 126)_ = 18.2; *p* = 1.2 x 10^−7^] and substrain [F_(2, 126)_ = 25.0; *p* = 7.1 x 10^−10^] differences among the three vendor-supplied DBA/2 substrains – DBA/2J, DBA/2NTac and DBA/2NCrl. Cocaine-induced locomotor activity on Day 3 was significantly higher than activity following saline administration on Days 1 and 2 (both *p* < 0.001; Figure 4A). DBA/2NTac mice were significantly less active than both DBA/2J and DBA/2NCrl (both *p* < 0.001). No significant sex differences were observed.

#### Bred In-house

The same set of DBA/2 mice were bred in-house at UNC and mice from the first generation were tested for cocaine-induced locomotor activity. As with the vendor-supplied DBA/2 substrains, we observed significant day [F_(2, 288)_ = 23.5; *p* = 3.6 x 10^−10^] and substrain (F_(2, 288)_ = 10.5; *p* = 3.8x10^−5^) effects. We also observed a significant day x substrain interaction [F_(4, 288)_ = 4.1; *p* = 0.003]. Although none of the substrains differed for locomotor activity on Day 1, DBA/2NTac mice had significantly lower locomotor activity than both DBA/2J (*p* = 0.014) and DBA/2NCrl (*p* = 0.028) mice on Day 2. DBA/2NTac mice also differed from DBA/2J (*p* = 0.003) and DBA/2NCrl (*p* = 0.002) mice on Day 3 (Figure 4B).

### Basal and Cocaine-Induced Locomotor Behavior Differs Across FVB/N Mice Bred In-house but Not the Vendor-Supplied Cohort

#### Vendor Supplied

**Figure 5:**
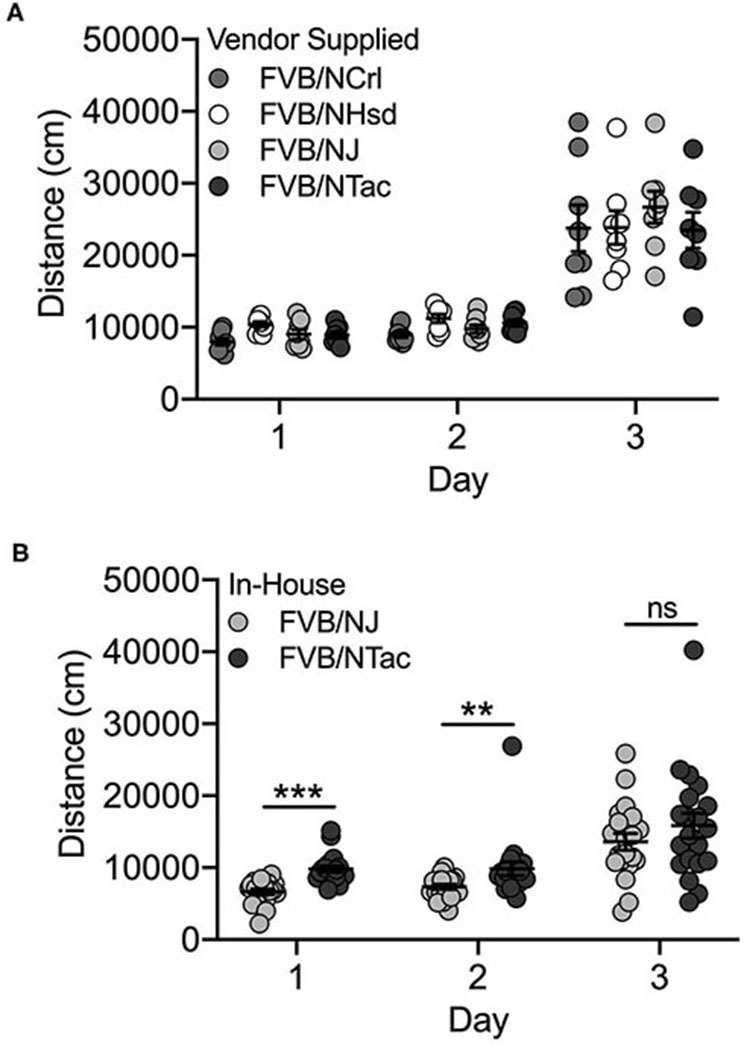
FVB/NJ and FVB/NTac substrains bred in-house differ for saline-induced locomotor activity on Days 1 and 2, but not cocaine-induced locomotor activation on Day 3. **(A)** Vendor-supplied FVB substrains are significantly activated in response to cocaine exposure on Day 3. FVB/NCrl mice are significantly less active than FVB/NHsd mice. **(B)** FVB/NTac mice bred in-house are significantly more active than FVB/NJ mice bred in-house following saline exposure on Days 1 and 2, but not following cocaine exposure on Day 3. Each data point represents an individual mouse, error bars are standard error of the mean. **p < 0.01; ***p < 0.001.

We identified significant day [F_(2, 72)_ = 207.8; *p* = 1.2 x 10^−30^] and substrain [F_(3, 72)_ = 4.7; *p* = 0.005] effects but no substrain by day interaction for the vendor-supplied FVB/N substrains. All FVB/N substrains showed significantly increased locomotor activity on Day 3 after exposure to cocaine (Figure 5A). FVB/NCrl mice are significantly less active than FVB/NHsd mice (*p* = 0.002; data not shown). No significant sex differences or interactions were observed.

#### Bred In-house

Significant day [F_(2, 107)_ = 35.7; p = 1.3 x 10^−12^], substrain [F_(2, 107)_ = 30.4; *p* = 2.5 x 10^−7^] and sex [F_(1, 107)_ = 4.7; *p* = 0.032] effects as well as substrain by day (F_(2, 107)_ = 3.5; *p* = 0.034) and substrain by sex [F_(1, 107)_ = 8.3; *p* = 0.005] interactions were observed for FVB/NJ and FVB/NTac substrains that were bred in-house for 1–2 generations. The two substrains differed significantly for locomotor activity on Days 1 [*t*(38) = 6.1; *p* = 3.4 x 10^−7^] and 2 [*t*(38) = 3.4; *p* = 0.002] but not cocaine-induced locomotor activity on Day 3 [*t*(38) = 0.706; *p* = 0.485] (Figure 5B). FVB/NJ females are significantly more active than FVB/NJ males (*t*(58) = 2.0; *p* = 0.045) but male and female FVB/NTac mice do not differ (*t*(44) = 0.943; *p* = 0.351) (data not shown).

FVB substrains in this cohort were tested by two different individuals and experimenter was a significant covariate in the ANOVA (*p* = 0.009). The experimenter effect reflects decreased locomotor activity following cocaine exposure on Day 3 as well as increased variability in data collected by one experimenter vs the other (data not shown).

### Locomotor Response to Cocaine Did Not Differ Across 2 NOD Substrains

#### Bred In-house

**Figure 6:**
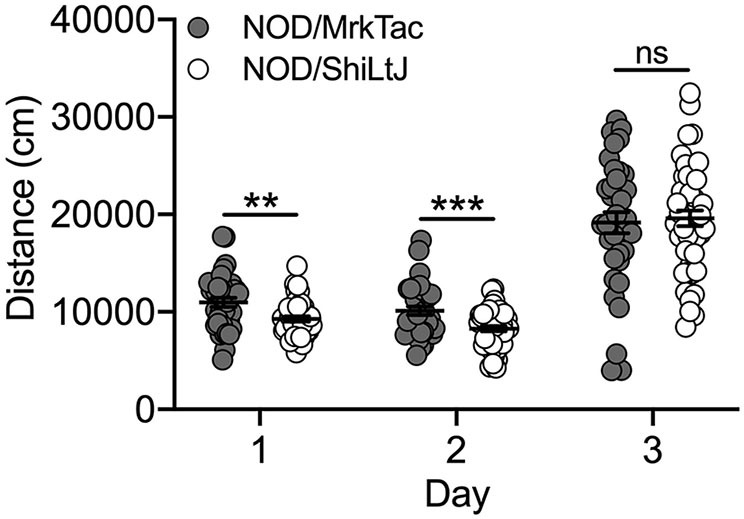
NOD substrains bred in-house differ for locomotor activity in response to saline but not cocaine. All NOD mice, regardless of substrain, had higher locomotor activity following cocaine administration compared to saline. NOD/MrkTac mice are significantly more active than NOD/ShiLtJ mice on Days 1 and 2 following saline exposure but not in response to cocaine on Day 3. Each data point represents an individual mouse, error bars are standard error of the mean. **p < 0.01; ***p < 0.001.

Characterization of cocaine-induced locomotor activation in NOD substrains was limited to those that were bred in-house at UNC. We observed a significant effect of both substrain [F_(1, 239)_ = 4.2; *p* = 0.04] and day [F_(2, 239)_ = 139.0; *p* = 9.0 x 10^−41^] as well as a significant substrain by day interaction [F_(2, 239)_ = 5.1; *p* = 0.007]. NOD/MrkTac mice were significantly more active than NOD/ShiLtJ mice on Days 1 (*t*(82) = 3.0; *p* = 0.003) and 2 (*t*(82) = 3.5; *p* = 7.7 x 10–4) but not following cocaine exposure on Day 3 (Figure 6). No significant sex differences were detected.

## Discussion

Laboratory mice are invaluable tools in biomedical research and have contributed greatly to our understanding of biological and disease processes. Inbred strains, in particular, have been used for decades in studies aimed at identifying genes that contribute to behavioral phenotypes, including responses to various drugs of abuse (27, 39–41). These studies have been very successful in identifying chromosomal regions that likely harbor causal genetic variants. However, the genetic diversity present in mapping crosses between any two standard inbred strains and the sheer number of potential causal genes and polymorphisms in mapped loci has hindered progress. Thus, these strategies rarely progress to identifying a specific causal gene or variant. The reduced genetic complexity in inbred mouse substrains offers the opportunity to overcome this hurdle and more rapidly and efficiently identify the causative gene and specific genetic variant.

In order to use the RCC approach to identify causal genes and genetic variants, one needs to identify substrains that exhibit phenotypic differences in the trait of interest. This approach has been used successfully to identify the *Cyfip2* gene as a regulator of basal and cocaine-induced locomotor activity, behavioral sensitization and binge-eating in two C57BL/6 substrains, C57BL/6J and C57BL/6NJ (34, 42). The study of addiction-related behaviors, and specifically initial locomotor sensitivity to psychostimulants, has been mostly limited to the C57BL/6 inbred substrains. We assessed differences in cocaine-induced locomotor response across 20 inbred mouse substrains from 6 different strains. Our data represent the first behavioral characterization of cocaine-induced locomotor activation in most of these substrains.

Two sets of strains showed particularly robust substrain differences that were replicated across experimental cohorts. C3H/HeNTac mice had significantly higher basal and cocaine-induced locomotor activity than C3H/HeNCrl and C3H/HeNHsd mice in one experimental cohort and C3H/HeJ mice in both cohorts (Figures 3A,B). The similarity of the behavioral phenotype in C3H/HeJ, C3H/HeNCrl and C3H/HeNHsd substrains suggests that the causal variant(s) may have become fixed in the C3H/HeNTac substrain after it diverged from the other C3H/HeN lines in 1974 (C3H/HeNCrl) and 1983 (C3H/HeNHsd). However, we must also consider the possibility that C3H/HeNCrl and C3H/HeNHsd behavioral phenotypes result from a different variant or variants. We also observed consistent substrain differences in behavior in DBA/2 mice that were supplied by commercial vendors and bred in-house. DBA/2NTac mice had significantly lower basal and cocaine-induced locomotor activity compared to DBA/2J and DBA/2NCrl in both cohorts (Figures 4A,B). These data suggest that the causal variant(s) likely arose in the DBA/2NTac substrain after it diverged from DBA/2N in 1981.

Correspondence of significant substrain differences across the two cohorts suggests a strong genetic component and supports the use of the RCC to identify specific causal variants that influence basal locomotor activity and response to cocaine. Our observation of significant substrain differences in basal and/or cocaine-induced locomotor behavior in all of the strain sets examined (Supplementary Table 2) does suggest that a complex genetic landscape underlies these behaviors. The presence of more than one causal polymorphism and/or background genetic factors that contribute to behavioral differences may make it necessary to produce larger mapping crosses that are adequately powered to detect QTL.

We also identified different behavioral phenotypes in vendor-supplied substrains vs. those bred in-house. For example, FVB/NTac mice bred in-house were significantly more active than FVB/NJ mice bred in-house across all three days of testing (Figure 5B) whereas vendor-supplied FVB substrains showed similar locomotor behavior across all three test days (Figure 5A). Neither A/J nor A/JOlaHsd substrains bred in-house were significantly activated in response to cocaine and in fact, locomotor behavior in these two substrains decreased significantly across all 3 days of testing (Figures 1B,C). Similarly, vendor-supplied A/J and A/JOlaHsd substrains were not significantly activated in response to cocaine, but we observed no significant decrease in locomotor activity across test days (Figure 1A). It is important to highlight that cross-cohort comparisons, especially those highlighting behavioral differences, must be made with caution as these differences may be driven in part by confounding factors.

The availability of cohoused C3H/HeNTac and C3H/HeJ substrains vs those maintained in substrain-specific housing allows us to examine indirect genetic effects that might influence basal and cocaine-induced locomotor activity. Indirect genetic effects are environmental effects that result from the genetic background of interacting conspecifics (43, 44). We tested the hypothesis that C3H/HeJ mice housed in mixed substrain cages were behaviorally different than C3H/HeJ mice from substrain-specific cages (and similarly for C3H/HeNTac). Our analyses yielded no significant effect of housing demonstrating that cage-level interactions among C3H/He substrains did not contribute to behavioral differences.

The observation of behavioral differences in the same substrain based on the source from which mice were obtained suggests that other environmental factors could be responsible. Multiple studies have systematically examined environmental factors that might affect behavioral phenotypes including, but not limited to diet, type of cage, cage density, season, time of day, transportation and experimenter effects (45–49). However, previous studies have generally assessed behavioral differences in mice tested across multiple sites. We examined behavior in all mice, independent of the source, in the same behavioral facility (and same testing room) at UNC. As such, we were able to control, to the extent possible, the environment to which the mice were exposed in the 5-week period leading up to testing. Mice were maintained on the same light cycle, tested during the same time of day, provided the same diet and water and housed in the same caging and animal holding room prior to and throughout testing.

The stress of transportation is an obvious difference between vendor-supplied mice and those bred in-house. We don't believe transportation stress could fully explain behavioral differences between mice from different sources. Previous studies have shown that transportation has very little effect on behavioral outcomes (45, 48). Moreover, vendor-supplied mice arrived at UNC very close to weaning age and were habituated to our vivarium conditions for approximately 5 weeks prior to testing.

Experimenter effects can also have an impact on behavioral outcomes. All mice supplied directly from the vendor were tested by the same animal handler (a female), whereas substrains bred in-house were tested by a group of 5 animal handlers including males and females. At least two studies have established that experimenter effects (49) and even the sex of the individual testing the mice (47) can significantly affect the outcome of behavioral tests. Although not widely observed, our analyses support significant behavioral differences due to experimenter in two substrain cohorts. However, drawing broader conclusions from these results is confounded by experimental parameters. For example, experimenters were not distributed evenly across all batches for all substrains and although we tried to balance substrains across all test batches, the composition of substrains included in each batch varied such that experimenter differences might also reflect substrain differences.

The gut microbiome has been implicated in numerous behavioral traits including locomotor response to psychostimulants (50, 51). Composition of the gut microbiota, even in the same inbred strain background, can vary across time, from vendor to vendor, and even between different facilities and animal holding rooms at the same vendor or institution (52, 53). These differences can be attributed to a host of environmental factors including diet, caging, bedding and water supply (54–56). Host genetic background also plays a significant role in the composition of the gut microbiota (57). Profound or even subtle changes in the gut microbiota in response to relocation from vendors to our vivarium could interact with different genetic backgrounds to significantly impact behavior. The relationship between genetic background and behavior becomes even more complicated when one considers that substrain behaviors attributable to stable differences in the gut microbiota could be erroneously ascribed solely to genetics. Shifts in the gut microbiota in response to changing environments could alter phenotypes and impact replicability from study to study. Recent studies have also established that the maternal microbiome can affect offspring neurodevelopment and impact behavior in adulthood (58–60). Thus, it is important to consider not only the source of the mice being tested, but the composition of the maternal microbiome during neurodevelopment.

Finally, an important caveat of our study is the limitation of using a single, acute dose of 20 mg/kg cocaine. These data represent a first step for future work involving a more in-depth behavioral analyses of these substrains. Testing at additional doses is certainly warranted as dose dependent effects may reflect differences in drug sensitivity. It is also important to examine activity across the session as substrain differences in timing of the response, shifts in peak cocaine-induced locomotor activation and other behavioral patterns may not be adequately captured by collapsing across the entire 30-min session (Supplementary Figure 1). It is also important to note that dose-specific differences in cocaine sensitivity may not be fully captured using locomotor activity as the primary behavioral measure. Other behaviors, such as stereotypy, should also be assessed in an expanded range of doses. Finally, a full pharmacokinetic profile of these substrains will be essential in determining potential differences in drug metabolism that may be responsible for any observed behavioral differences.

In summary, this study expands the knowledge of phenotypic differences in locomotor activity and initial response to cocaine in 6 sets of inbred mouse substrains which had previously not been characterized. All six strain lineages displayed substrain differences in either basal- or cocaine-induced locomotor behavior and can be utilized in RCCs to identify causal genetic variants. Expanded behavioral testing in these substrains to characterize the rewarding and reinforcing effects of cocaine is an important next step. Environmental factors also warrant follow-up, as differences in behavior were observed across the same inbred substrains obtained from different sources. Substrains from C3H/He and DBA/2 lineages demonstrated stable and robust differences in cocaine-induced locomotor behavior, and are good candidates for additional studies to investigate genetic and environmental factors that contribute to initial cocaine sensitivity. Future studies can utilize these data to increase our understanding of the complex factors that increase CUD and potentially lead to new therapeutic targets.

## Data Availability Statement

The raw data supporting the conclusions of this article will be made available by the authors, without undue reservation.

## Ethics Statement

The animal study was reviewed and approved by UNC Institutional Animal Care and Use Committee.

## Author Contributions

CG, SS, MF, FP-M, and LT: conception and design of work and revising/editing manuscript. CG, SS, JF, DL, and LA-C: data collection. CG, SS, and LT: data analysis and drafting the manuscript. CG, SS, JF, DL, LA-C, GS, DM, MF, FP-M, and LT: approval of manuscript. SS, GS, DM, MT, FP-M, and LT: provided resources. All authors contributed to the article and approved the submitted version.

## Funding

This study was supported by pilot funding from NIDA P50 039841 to SS and LT. Pilot funding was used to purchase research materials and support personnel conducting studies. Funding from R21 DA052171 to LT was used to support personnel conducting studies and purchase research material. U24 HG010100, U19 AI100625, and P01 AI132130 to FP-M and MF provided funding to maintain inbred mouse substrain colonies that provided research subjects for the work.

## Conflict of Interest

The authors declare that the research was conducted in the absence of any commercial or financial relationships that could be construed as a potential conflict of interest.

## Publisher's Note

All claims expressed in this article are solely those of the authors and do not necessarily represent those of their affiliated organizations, or those of the publisher, the editors and the reviewers. Any product that may be evaluated in this article, or claim that may be made by its manufacturer, is not guaranteed or endorsed by the publisher.
