# Supplementary material for: Cocaine-Induced Locomotor Activation Differs Across Inbred Mouse Substrains
Source: Front Psychiatry. 2022 May 6;13:800245. doi: 10.3389/fpsyt.2022.800245 (PMC9120424; doi:10.3389/fpsyt.2022.800245)
Supplement: Supplementary file 1 [file Data_Sheet_1.PDF]

Supplemental Table 1

Strain	Substrain	Vendor	Strain Catalog Number	Origin	Cage Environment	Female	Male	Total	Females Day 1 Mean	Females Day 1 StdDev	Females Day 2 Mean	Females Day 2 StdDev	Females Day 3 Mean	Females Day 3 StdDev	Males Day 1 Mean	Males Day 1 StdDev	Males Day 2 Mean	Males Day 2 StdDev	Males Day 3 Mean	Males Day 3 StdDev
A/J	A/J	Jackson Laboratory	#000646	Bred in-house	Cohoused	20	17	37	1432.8	891.1	991.8	764.3	523.1	451.2	1817.5	942.8	1247.8	756.9	903.9	1251.5
	A/JOlaHsd	Envigo	#049	Bred in-house	Cohoused	19	17	36	824.8	597.2	383.4	472.2	190.6	390.4	1087.0	772.7	727.7	774.2	441.1	1029.9
	A/J	Jackson Laboratory	#000646	Vendor Supplied	Not cohoused	8	8	16	1986.1	1030.5	1561.4	788.0	381.8	554.8	1125.5	659.5	1365.6	744.4	1312.2	1813.7
	A/JCr	Charles River Laboratories	#563	Vendor Supplied	Not cohoused	8	8	16	1677.3	796.0	1778.3	860.7	3710.4	4200.6	1957.7	1044.4	1338.9	739.9	2759.4	4899.7
	A/JOlaHsd	Envigo	#049	Vendor Supplied	Not cohoused	8	8	16	754.6	452.5	780.7	599.8	1244.1	2005.9	1032.9	580.1	1121.6	662.6	1261.1	1687.8
BALB/c	BALB/cByJ	Jackson Laboratory	#001026	Bred in-house	Not cohoused	4	2	6	7217.4	3571.3	5257.9	2922.1	4278.9	3984.0	4709.8	29.6	81.8	46.7	880.0	810.5
	BALB/cJ	Jackson Laboratory	#000651	Bred in-house	Not cohoused	6	0	6	6611.2	5270.8	5056.4	6427.4	5597.2	6249.3						
	BALB/cAnNCrl	Charles River Laboratories	#028	Vendor Supplied	Not cohoused	7	8	15	3378.9	1917.6	1363.6	1236.4	1312.0	1713.1	3641.1	1786.0	911.8	803.5	2770.1	4465.3
	BALB/cAnNHsd	Envigo	#047	Vendor Supplied	Not cohoused	8	8	16	3893.3	1787.8	1982.2	1790.5	1784.7	1901.5	3017.1	1613.5	1542.8	1227.8	1511.0	3293.0
	BALB/cByJ	Jackson Laboratory	#001026	Vendor Supplied	Not cohoused	7	8	15	7515.4	2737.0	3970.4	3288.1	10821.6	7551.5	3975.8	2669.8	3218.4	2961.9	7690.1	8439.3
	BALB/cJ	Jackson Laboratory	#000651	Vendor Supplied	Not cohoused	8	8	16	6389.7	2109.3	4089.8	2679.2	6701.4	6789.9	5612.9	1781.9	2149.3	1844.8	2723.9	3765.6
C3H/He	C3H/HeJ	Jackson Laboratory	#000659	Bred in-house	Cohoused	11	8	19	3305.0	810.6	3750.0	1603.1	18080.8	5727.2	3528.4	1490.3	3826.3	1641.8	18911.4	5873.4
	C3H/HeNTac	Taconic	C3H-F/C3H-M	Bred in-house	Cohoused	12	8	20	5669.8	831.2	6113.1	1603.7	36004.9	8427.3	5471.8	1854.7	5825.8	1964.7	26960.6	12045.8
	C3H/HeJ	Jackson Laboratory	#000659	Bred in-house	Not cohoused	18	14	32	3398.7	1057.5	3511.2	1472.4	14617.3	5142.1	3498.3	852.1	3333.9	903.4	15314.5	6038.8
	C3H/HeNTac	Taconic	C3H-F/C3H-M	Bred in-house	Not cohoused	21	16	37	6019.4	2047.1	7199.9	2008.3	27739.8	11743.4	6905.3	1561.1	6720.5	1939.6	27654.4	10271.4
	C3H/HeNHsd	Envigo	#040	Bred in-house	Not cohoused	32	32	64	4112.8	1432.8	4850.3	1469.5	19374.4	7991.4	3704.0	1677.0	3999.2	1716.9	14856.2	8853.0
	C3H/HeNCrl	Charles River Laboratories	#025	Bred in-house	Not cohoused	19	32	51	3380.1	1763.3	3714.1	2018.8	17761.2	11014.1	3738.6	1293.0	4479.2	1203.3	19576.2	7152.1
DBA/2	DBA/2J	Jackson Laboratory	#000671	Bred in-house	Cohoused	20	18	38	5925.7	1120.5	5204.3	1959.3	13877.4	10429.2	5447.7	2101.7	4738.7	1931.9	12517.9	7447.5
	DBA/2NCrl	Charles River Laboratories	#026	Bred in-house	Cohoused	11	19	30	6339.9	2085.8	5358.3	2046.3	15088.0	9565.1	6016.1	1122.3	4511.9	1370.8	11203.1	7388.3
	DBA/2NTac	Taconic	DBA2-F/DBA2-M	Bred in-house	Cohoused	19	15	34	5569.3	2062.3	3354.5	1759.7	6537.0	5796.6	6507.0	1475.8	4418.3	1336.9	5633.1	3263.9
	DBA/2J	Jackson Laboratory	#000671	Vendor Supplied	Not cohoused	8	8	16	3787.3	2000.2	4129.7	2020.1	8043.1	6835.0	3264.8	1192.3	4900.4	788.0	12137.8	5662.2
	DBA/2NCrl	Charles River Laboratories	#026	Vendor Supplied	Not cohoused	8	8	16	4543.2	2391.9	4179.7	2357.3	12003.3	12577.2	3560.4	1490.8	3132.5	1829.8	11248.2	4662.4
	DBA/2NTac	Taconic	DBA2-F/DBA2-M	Vendor Supplied	Not cohoused	8	8	16	2519.2	1805.3	1426.6	1263.5	4456.6	4490.4	2145.4	2331.9	1580.9	1743.0	2178.1	2156.5
FVB/N	FVB/NJ	Jackson Laboratory	#001800	Bred in-house	Cohoused	11	9	20	7476.6	797.6	7990.5	1636.5	14784.8	4757.5	5606.9	1921.9	6537.1	1120.4	12139.6	5758.2
	FVB/NTac	Taconic	FVB-F/FVB-M	Bred in-house	Cohoused	11	9	20	9687.1	972.8	9504.4	1436.5	12803.6	5148.7	9931.8	2883.1	10260.0	6354.3	19566.4	9029.5
	FVB/NCrl	Charles River Laboratories	#207	Vendor Supplied	Not cohoused	4	4	8	8405.1	1304.0	9549.8	1398.9	26917.5	8366.8	7585.5	1498.5	8372.8	143.6	20593.8	9867.8
	FVB/NHsd	Envigo	#118	Vendor Supplied	Not cohoused	4	4	8	9899.0	1290.6	10596.0	1884.7	25221.5	8730.3	10881.7	694.4	11859.5	1448.5	22500.1	4567.3
	FVB/NJ	Jackson Laboratory	#001800	Vendor Supplied	Not cohoused	4	4	8	8562.1	2309.6	9537.4	2154.4	26447.7	9389.4	9488.7	1510.0	9968.9	929.0	26892.3	1669.2
	FVB/NTac	Taconic	FVB-F/FVB-M	Vendor Supplied	Not cohoused	4	4	8	9682.4	1257.9	11098.1	1430.5	19374.7	5623.9	8162.8	733.0	10074.8	1049.7	27565.5	6272.4
NOD	NOD/MrkTac	Taconic	NOD-F/NOD-M	Bred in-house	Cohoused	18	19	37	10695.1	3146.5	11021.8	2319.8	19816.5	7952.8	11257.3	2545.6	9242.7	2770.4	18533.8	4969.6
	NOD/ShiLtJ	Jackson Laboratory	#001976	Bred in-house	Cohoused	18	29	47	9133.8	1561.9	8923.1	1766.1	21956.3	4947.3	9369.3	1937.1	7865.4	1886.6	18134.8	5490.8

Supplemental Table 2

	Substrain	Day	Sex	Substrain x Day	Substrain x Sex	Experimenter	Transformation	Lambda
A/J Vendor	1.90E-02					NA	Box Cox	Square Root
A/J In-House	1.41E-08	2.50E-12	9.00E-03			NS	Box Cox	Square Root
BALB/c Vendor	6.20E-08	3.90E-05	0.016			NA	Box Cox	Square Root
BALB/c In-House			0.043			NA	Box Cox	Square Root
DBA/2 Vendor/NonCohoused	7.12E-10	1.20E-07				NA	Box Cox	Square Root
DBA/2 In-House/Cohoused	3.80E-05	3.60E-10		0.003		NA	Box Cox	Natural Log
C3H/He In-House/NonCohoused	3.04E-16	4.70E-83			5.97E-08	2.00E-06	Box Cox	Natural Log
C3H/He In-House/Cohoused	7.91E-11	3.60E-40				NS	Box Cox	Natural Log
FVB/N Vendor	0.005	1.20E-30				NA	Box Cox	1/SquareRoot
FVB/N In-House	2.50E-07	1.30E-12	0.032	0.034	0.005	0.009	Rank Inverse Normal	NA
NOD In-House	0.042	9.00E-41		0.007		NA	Box Cox	Log

Supplemental Figure 1

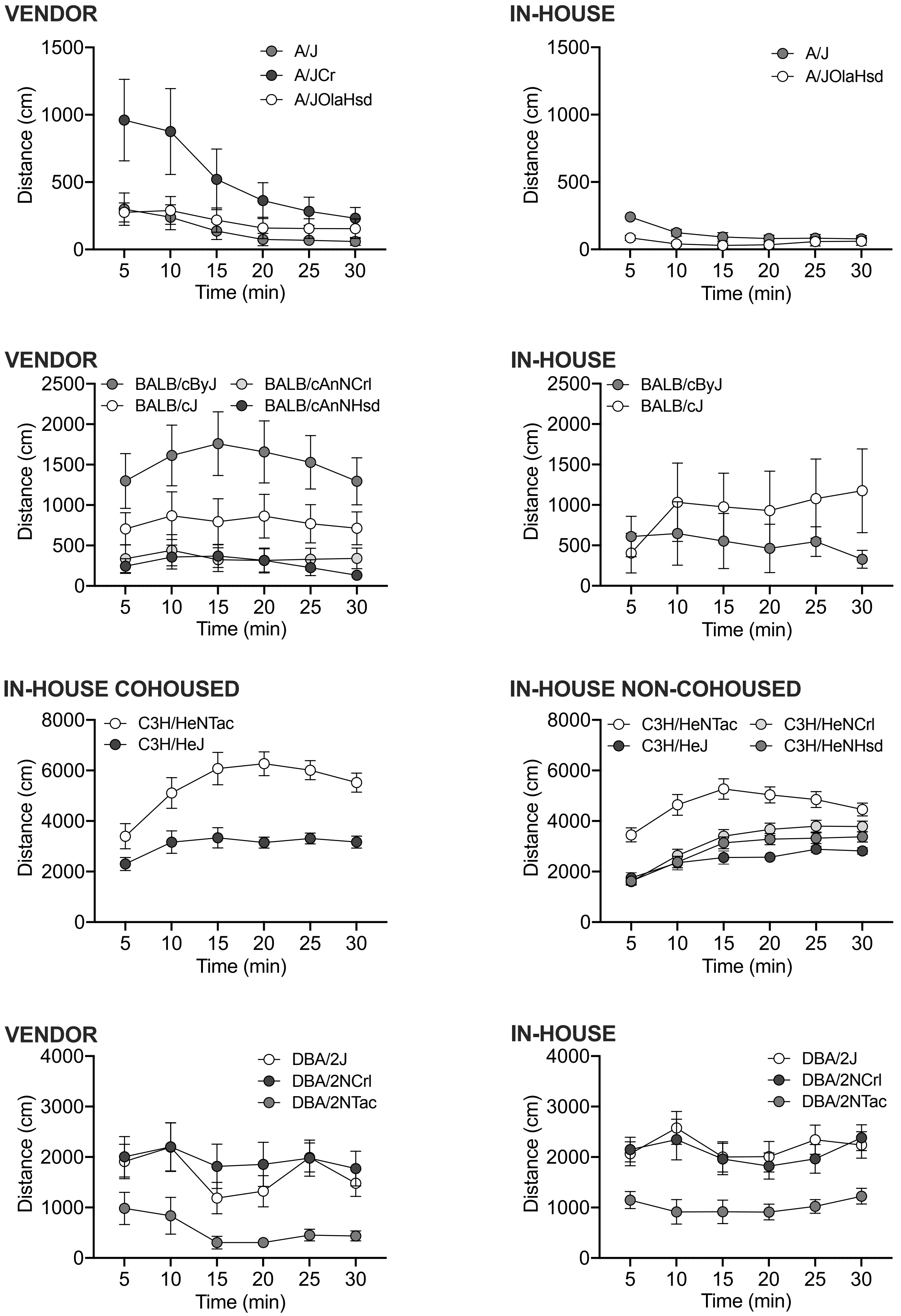

Supplemental Figure 1 - continued

VENDOR

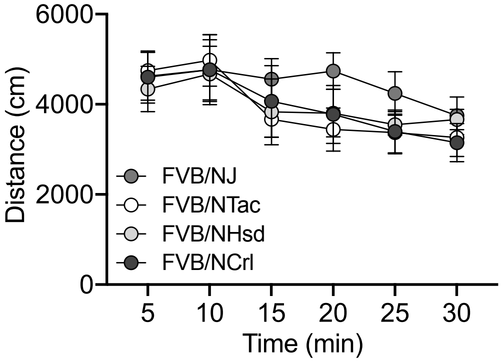

IN-HOUSE

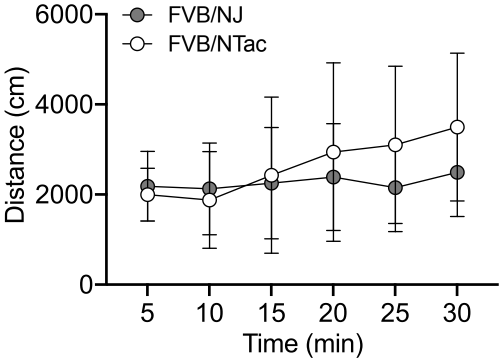

IN-HOUSE

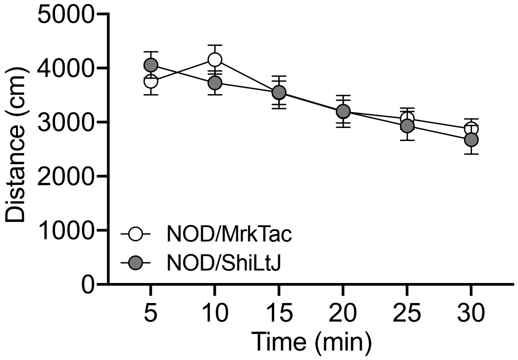

Figure legends

Supplemental Table 1.
Means and standard deviations for locomotor activity on each test day for all inbred mouse substrains.

Supplemental Table 2.
Summary of factors included in ANOVA analyses for inbred mouse substrains. Table includes Lambda used for datasets which required Box Cox transformation.

Figure 1
Cocaine induced locomotor activity shown in 5-min bins across 30-min testing session on Day 3 for each group of substrains.
